# Decellularized Human Skeletal Muscle as Biologic Scaffold for Reconstructive Surgery

**DOI:** 10.3390/ijms160714808

**Published:** 2015-07-01

**Authors:** Andrea Porzionato, Maria Martina Sfriso, Alex Pontini, Veronica Macchi, Lucia Petrelli, Piero G. Pavan, Arturo N. Natali, Franco Bassetto, Vincenzo Vindigni, Raffaele De Caro

**Affiliations:** 1 Section of Human Anatomy, Department of Molecular Medicine, University of Padova, Via Gabelli 65, Padova 35127, Italy; E-Mails: andrea.porzionato@unipd.it (An.P.); mariamartina.sfriso@yahoo.it (M.M.S.); veronica.macchi@unipd.it (V.M.); lucia.petrelli@unipd.it (L.P.); 2 Clinic of Plastic Surgery, University of Padova, Via Giustiniani 2, Padova 35127, Italy; E-Mails: alex.pontini@libero.it (Al.P.); franco.bassetto@unipd.it (F.B.); vincenzo.vindigni@unipd.it (V.V.); 3 Department of Industrial Engineering, University of Padova, Via G. Marzolo 9, Padova 35131, Italy; E-Mails: piero.pavan@unipd.it (P.G.P.); arturo.natali@unipd.it (A.N.N.)

**Keywords:** skeletal muscle, scaffold, decellularization, regenerative medicine, human, reconstructive surgery, extracellular matrix, stem cells, tissue engineering, scanning electron microscopy

## Abstract

Engineered skeletal muscle tissues have been proposed as potential solutions for volumetric muscle losses, and biologic scaffolds have been obtained by decellularization of animal skeletal muscles. The aim of the present work was to analyse the characteristics of a biologic scaffold obtained by decellularization of human skeletal muscles (also through comparison with rats and rabbits) and to evaluate its integration capability in a rabbit model with an abdominal wall defect. Rat, rabbit and human muscle samples were alternatively decellularized with two protocols: n.1, involving sodium deoxycholate and DNase I; n.2, trypsin-EDTA and Triton X-NH_4_OH. Protocol 2 proved more effective, removing all cellular material and maintaining the three-dimensional networks of collagen and elastic fibers. Ultrastructural analyses with transmission and scanning electron microscopy confirmed the preservation of collagen, elastic fibres, glycosaminoglycans and proteoglycans. Implantation of human scaffolds in rabbits gave good results in terms of integration, although recellularization by muscle cells was not completely achieved. In conclusion, human skeletal muscles may be effectively decellularized to obtain scaffolds preserving the architecture of the extracellular matrix and showing mechanical properties suitable for implantation/integration. Further analyses will be necessary to verify the suitability of these scaffolds for *in vitro* recolonization by autologous cells before *in vivo* implantation.

## 1. Introduction

Loss of skeletal muscle tissues may occur as a result of traumatic injuries, degenerative changes, infections or neoplasms. Clinically, restoration of large volumetric muscle loss involves transfer of autologous muscle tissue or muscle flaps from local or distant sites to the area of injury [1,2], although these procedures are associated with donor site morbidity resulting in functional loss and volume deficiency at the donor site. Conversely, although xenografts and allografts may eliminate donor-site morbidity and decrease operating time, they are associated with the risk of severe immune response, transmission of infective diseases and slower integration with native tissue [3].

Therefore, the development of engineered skeletal muscle grafts from homologous and autologous tissues has recently been proposed as a potential solution for replacement of volumetric muscle losses, in order to avoid donor site complications and permit the availability of large quantities of tissues with good histocompatibility. The rationale behind the use of native matrix materials is the isolation of extracellular matrix proteins that are site-specific and provide protein “footprints” of previous resident cells. Skeletal muscle is a highly organized and complex tissue, not only rich in contractile elements and connective tissue but also of vessels and nerve fibers. The extracellular matrix of skeletal muscle is known to play a pivotal role in muscle development and regeneration. It is a reservoir of growth factors regulating function and regeneration of muscle fibers. The process of constant interchange between cells and the extracellular matrix, described as dynamic reciprocity, determines cell fate and triggers the shift from proliferation to structure formation [4]. Furthermore, the muscle extracellular matrix takes part in mechanisms of cell migration, involving different precursors of muscle cells. Moreover, extracellular matrix proteins are among the most conserved proteins [5] and usually they do not elicit an immune response. Thus, the development of a muscle-derived scaffold, able to maintain the composition and integrity of the extracellular matrix, together with removal of all the cellular elements through decellularization protocols, has been proposed for muscle regeneration studies.

Biocompatible native extracellular matrix scaffolds can be generated by muscle tissues with decellularization protocols involving physical (e.g., freeze/thaw cycles), enzymatic (e.g., trypsin) and/or chemical (e.g., SDS) mechanisms [6,7]. Natural muscle-derived scaffolds have first been realized through the application of decellularization protocols to cardiac [8,9,10] and smooth [11,12] muscles. Following experimental studies developed decellularized scaffolds from animal skeletal muscle (e.g., [13,14,15,16,17]) and verified their suitability to repair muscle defects *in vivo* (e.g., [2,14,15,16,17,18,19]). Comparable results have also been reported in promoting functioning skeletal myogenesis, *in vitro* and *in vivo*, between scaffolds derived from skeletal and cardiac muscles [20]. An engineered composite tissue graft has also been recently proposed through decellularization of all components (bone, muscles, tendons, vessels and nerves) of rat and primate forearms and following recellularization with myoblasts, embryonic fibroblasts and human umbilical vein endothelial cells [21].

To the best of our knowledge, there are no reports in the literature regarding the development of decellularized scaffolds from human skeletal muscles. Thus, in the present study, we performed a comparative analysis between different decellularization protocols in rat, rabbit and human skeletal muscle samples. In the literature, there are contrasting results regarding the ability of decellularized (and not-recellularized) scaffolds to be integrated with regenerative response in a muscle defect, so that a trial of scaffold integration was performed in rabbits with a human-derived scaffold (in the absence of *in vitro* recellularization) in order to preliminarily evaluate its capability to be integrated, maintaining its connective structure without inflammatory rejection response, and its eventual regenerative potentiality, through proliferation, migration and differentiation of skeletal muscle precursors from surrounding muscle components.

## 2. Results

From a macroscopic point of view, decellularization of rat, rabbit and human skeletal muscles modified tissue characteristics of the samples. In particular, protocol 2 changed the sample color from reddish to whitish and slightly reduced its consistency. However, the muscle samples preserved their volume and homogeneity, without showing tissue ruptures and appeared mechanically suitable for surgical sutures. Tissue changes produced by protocol 1 were less evident, muscle samples maintaining a pink color suggestive of incomplete muscle cell removal.

**Figure 1:**
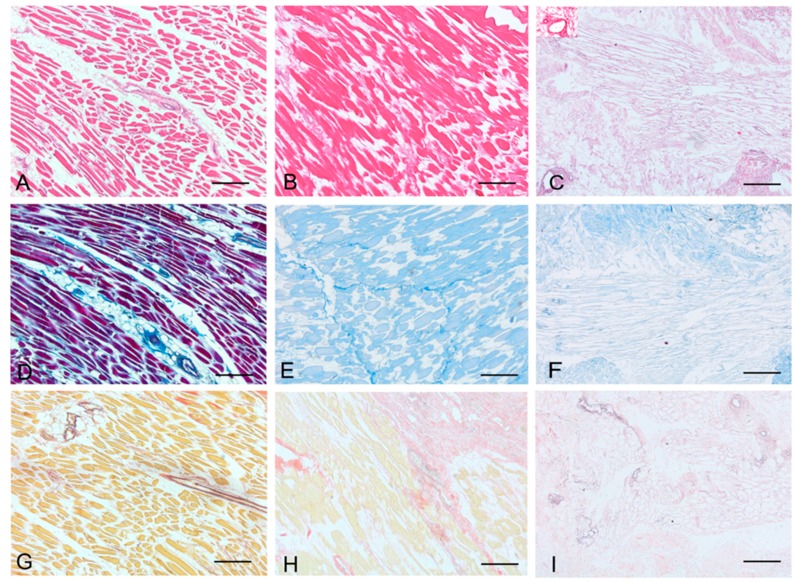
Representative sections of rat skeletal muscle stained with Hematoxylin & Eosin (**A**–**C**), Azan Mallory (**D**–**F**) and Van Gieson (**G**–**I**), before decellularization (**A**,**D**,**G**), after decellularization protocol 1 (**B**,**E**,**H**) and after protocol 2 (**C**,**F**,**I**), showing the higher effectiveness of protocol 2 in decellularization, with persistence of three-dimensional organization of collagen (**F**) and elastic (**I**) fibers. Scale bars: (**A**–**I**), 150 µm.

**Figure 2:**
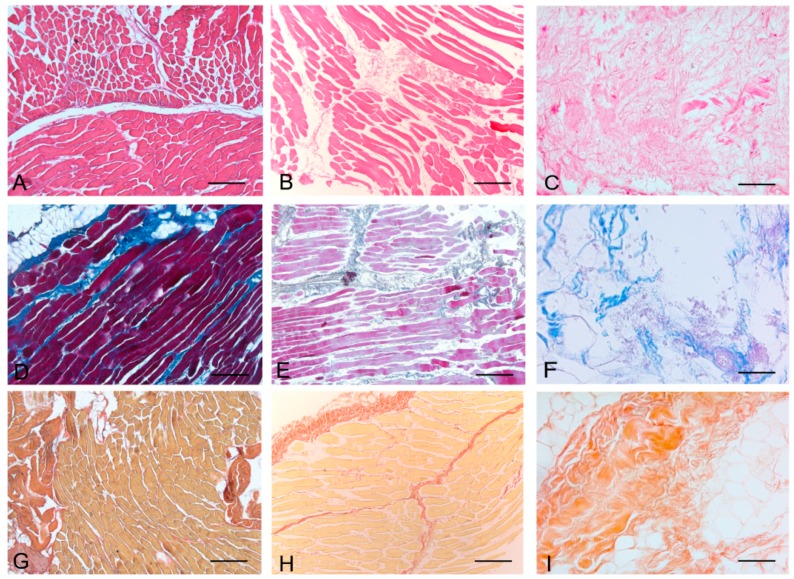
Representative sections of rabbit skeletal muscle stained with Hematoxylin & Eosin (**A**–**C**), Azan Mallory (**D**–**F**) and Van Gieson (**G**–**I**), before decellularization (**A**,**D**,**G**), after decellularization protocol 1 (**B**,**E**,**H**) and after protocol 2 (**C**,**F**,**I**), showing the higher effectiveness of protocol 2 in decellularization, with persistence of three-dimensional organization of collagen (**F**) and elastic (**I**) fibers. Scale bars: (**A**–**I**), 150 µm.

**Figure 3:**
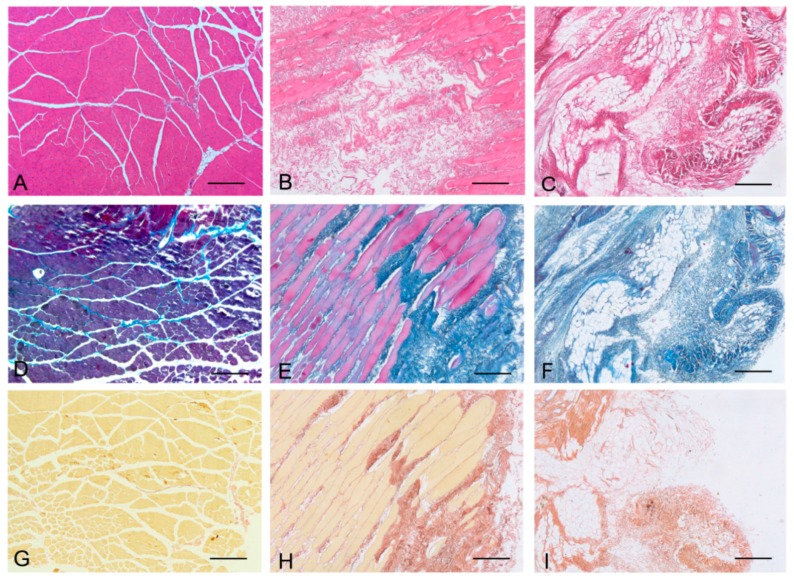
Representative sections of human skeletal muscle stained with Hematoxylin & Eosin (**A**–**C**), Azan Mallory (**D**–**F**) and Van Gieson (**G**–**I**), before decellularization (**A**,**D**,**G**), after decellularization protocol 1 (**B**,**E**,**H**) and after protocol 2 (**C**,**F**,**I**), showing the persistence of three-dimensional organization of collagen (**E**,**F**) and elastic fibers (**H**,**I**) in decellularized samples; but also incomplete removal of skeletal muscle fibers after decellularization protocol 1. Scale bars: (**A**–**I**), 150 µm.

**Figure 4:**
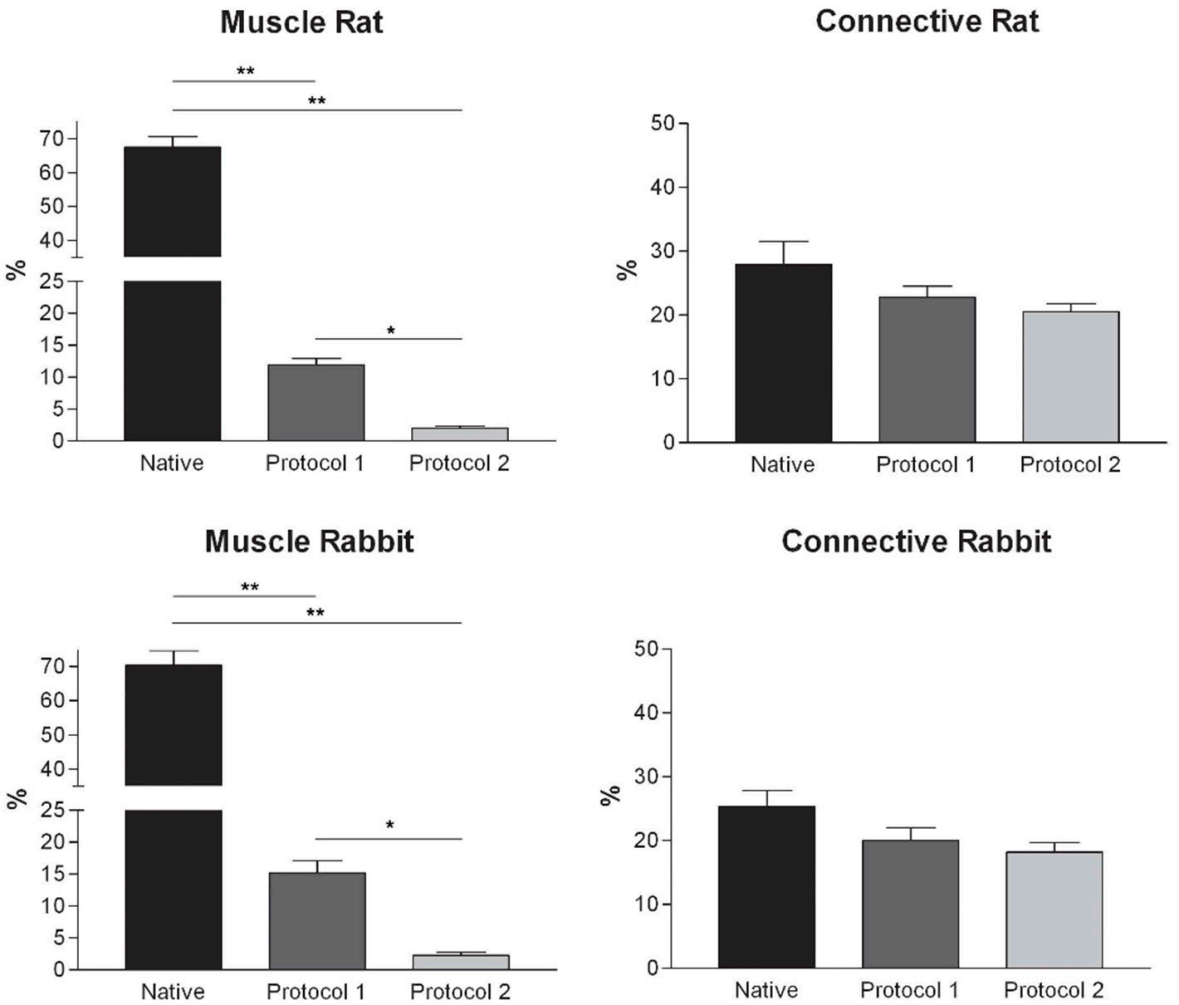

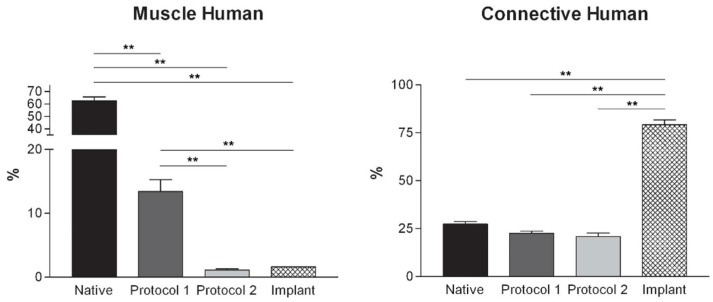
Graphs showing the percentages of muscle fiber content and connective tissue in muscle samples exposed to different decellularization protocols and, for human samples, after implants. The above two tissue components were morphometrically evaluated in sections stained with Azan-Mallory, muscle fibers and connective tissue staining in red/violet and blue, respectively. (*, *p <* 0.01; **, *p <* 0.001).

Histological stainings also demonstrated different efficiency by protocols 1 and 2 in removing cell materials. Skeletal muscle samples treated with protocol 1 showed persistence of myofibrills in some muscle fibers, although nuclei were no longer appreciable. Protocol 2, instead, completely removed skeletal muscle fibers in all the species considered, being no longer visible cell nuclei or myofibrillar elements. Conversely, decellularization protocols preserved the three-dimensional architecture of the extracellular matrix of skeletal muscle. Azan-Mallory and van Gieson stainings demonstrated the persistence of collagen and elastic fibers, respectively, in the network of extracellular matrix around spaces previously occupied by skeletal muscle fibers (Figure 1, Figure 2 and Figure 3). Morphometric analyses on sections stained with Azan-Mallory showed a significant reduction in muscle fiber content (red-staining) with both decellularization protocols in all the species investigated. Moreover, protocol 2 produced a further significant reduction with respect to protocol 1 in all the species (Figure 4). As it regards the connective component (blue-staining in Azan-Mallory), significant changes were not found (Figure 4).

**Figure 5:**
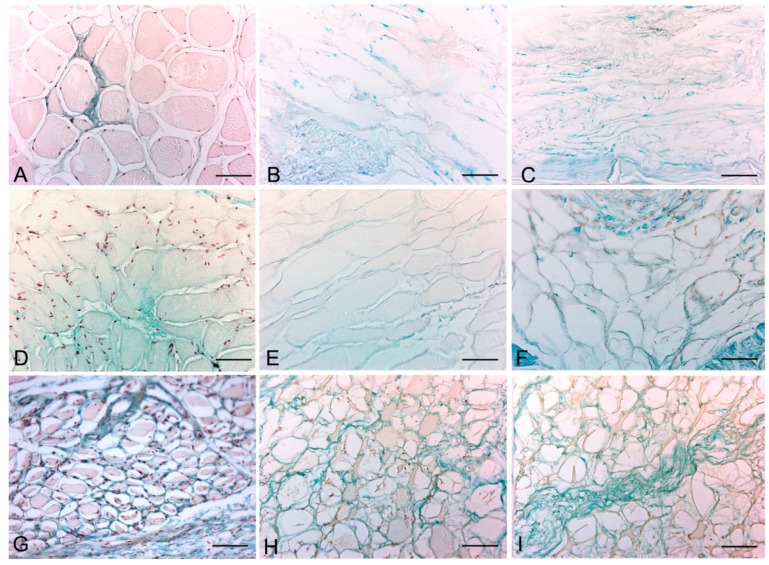
Representative sections of rat (**A**–**C**); rabbit (**D**–**F**) and human (**G**–**I**) skeletal muscle; before decellularization (**A**,**D**,**G**); after decellularization protocol 1 (**B**,**E**,**H**) and after protocol 2 (**C**,**F**,**I**); showing the persistence of blue-staining glycosaminoglycans in both decellularization protocols (Alcian blue, pH 2.5); Scale bars: (**A**–**I**): 75 µm.

Alcian blue staining at pH 2.5 and pH 1.0 revealed the persistence of all glycosaminoglycans and, in particular, sulfated glycosaminoglycans, respectively, after the decellularization (Figure 5 and Figure S1). Decellularization also preserved vessel profiles in all species samples: lumens remained visible with preservation of the vessel walls and their collagen and elastic components. Conversely, nuclei of endothelial and smooth muscle cells were no longer visible (Figure 1B).

**Figure 6:**
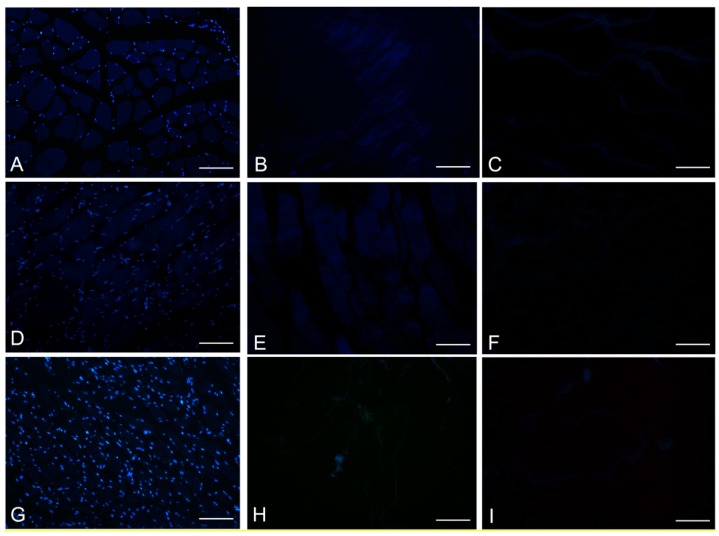
DAPI staining of rat (**A**–**C**); rabbit (**D**–**F**) and human (**G**–**I**) skeletal muscles, before decellularization (**A**,**D**,**G**); after decellularization protocol 1 (**B**,**E**,**H**) and after protocol 2 (**C**,**F**,**I**), showing loss of nuclear material in both protocols; Scale bars: (**A**–**I**), 150 µm.

**Figure 7:**
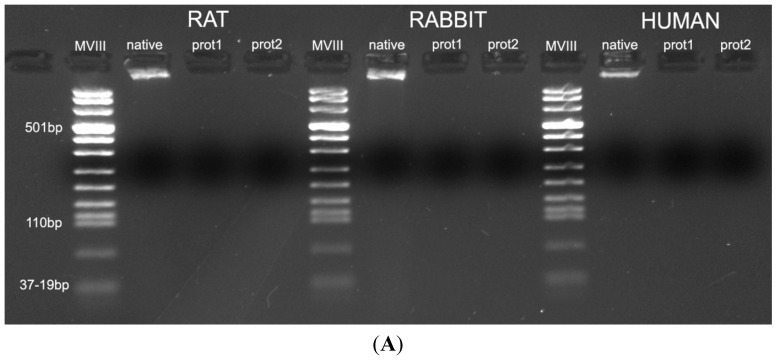

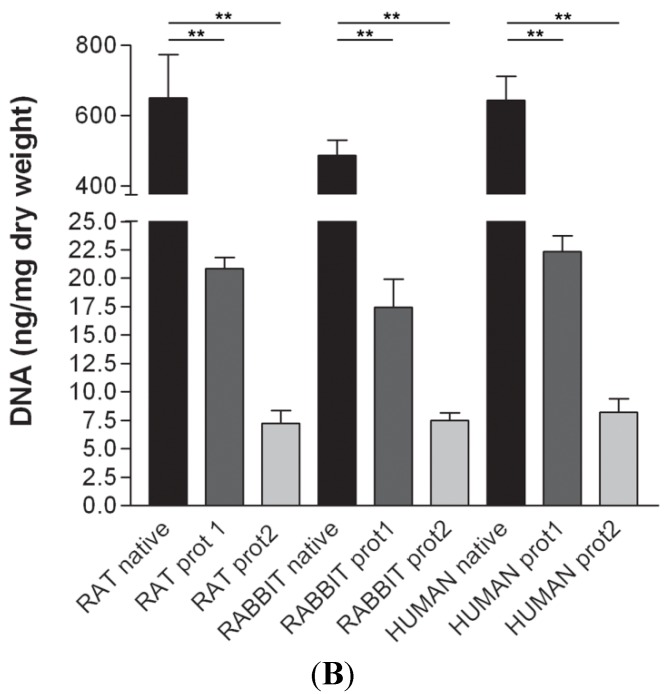
(**A**) Gel electrophoresis of DNA isolated from rat, rabbit and human skeletal muscles before decellularization (native) and after decellularization protocols. No traces of DNA were detectable with either protocols, confirming the effectiveness in removing the DNA component. (MVIII: DNA Molecular Weight Marker VIII; Roche Life Sciences, USA); (**B**) DNA contents of rat, rabbit and human skeletal muscles exposed to different decellularization protocols. (**, *p <* 0.001).

DAPI staining also showed complete removal of nuclear components in rat, rabbit and human scaffolds with both protocols (Figure 6). Quantitative evaluation of DNA by spectrophotometry demonstrated a significant reduction of DNA content with both protocols and in all the species (Figure 7). In particular, quantities of DNA less than 25 ng dsDNA per mg dry weight and less than 10 ng dsDNA per mg dry weight were found with protocols 1 and 2, respectively, in all the trials. These values are perfectly in accordance with the values reported in literature which indicate that optimal decellularization is achieved by <50 ng dsDNA per mg dry weight [22,23,24,25]. Moreover, in literature, residual DNA is usually considered of no concern if consisting of fragments less than 300 bp in length [26]. Thus, we also performed electrophoresis, but, in our experiments, the quantity of remaining dsDNA was so scarce, it was almost undetectable with both protocols and in all the species (Figure 7).

**Figure 8:**
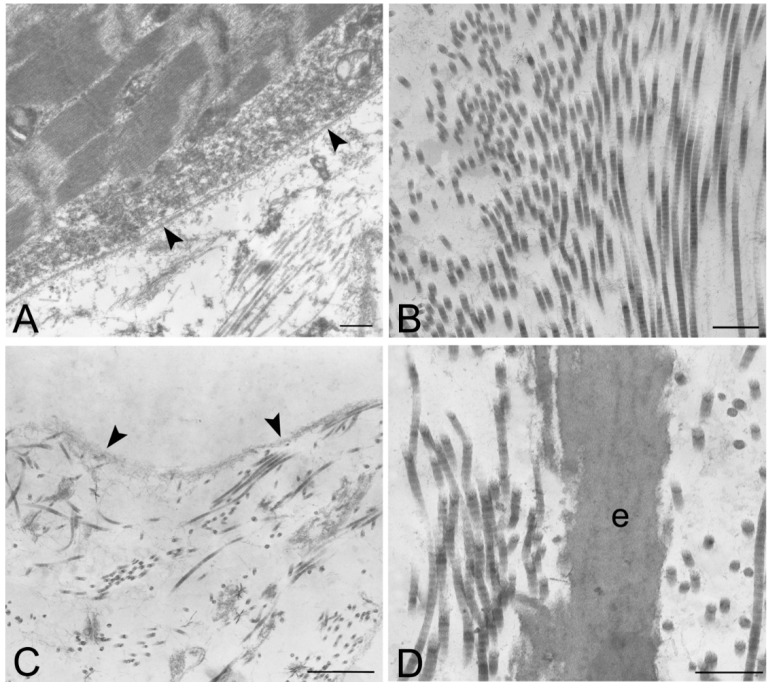
Transmission electron microscopy of human native skeletal muscle (**A**) and human skeletal muscles after decellularization protocols 1 (**B**) and 2 (**C**,**D**); Note the preservation of basal lamina (arrowheads in **A**,**C**) of muscle fibers, and the maintenance of the ultrastructure of collagen and elastic fibers (e) in the extracellular matrix; Scale bars: (**A**–**D**), 0.5 µm.

TEM demonstrated the preservation of the ultrastructure of collagen, elastic fibers and proteoglycans after decellularization, in particular in rat, rabbit and human samples treated with protocol 2 (Figure 8 and Figure S2). Compared to original biologic materials, all scaffolds showed an ordered distribution of collagen fibers with the characteristic banding still recognizable. The filamentous components of proteoglycans, anchoring the fibers to each other, were also still visible. The ultrastructure of elastin was also maintained, indicating that the decellularization protocols did not alter the elastic fibers during the treatment. Moreover, preservation of the basal lamina of skeletal muscle fibers was also demonstrated with TEM (Figure 7A,B).

**Figure 9:**
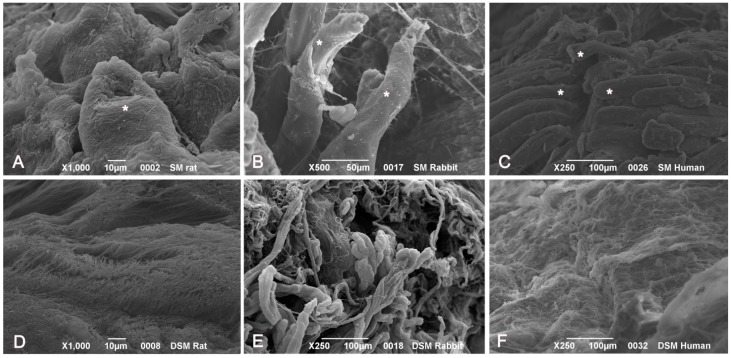

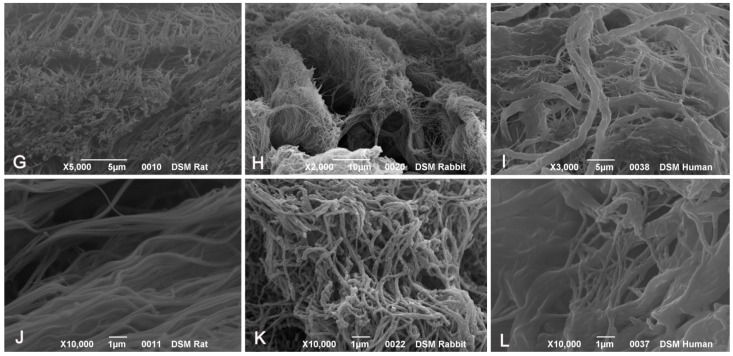
Scanning electron microscopy of muscle tissues, before decellularization (**A**–**C**), after decellularization protocol 1 (**D**–**F**) and after protocol 2 (**G**–**L**), taken from rats (**A**,**D**,**G**,**J**), rabbits (**B**,**E**,**H**,**K**) and humans (**C**,**F**,**I**,**L**). In native samples, myofibers (*) are visible surrounded by collagen fibers (**A**–**C**). In protocol 2 decellularization, muscle cells are no longer visible whereas the collagenic fibers of perimysium and endomysium still persist; in protocol 1 decellularization, some components of muscle fibers are still present. Scale bars: (**C**,**E**,**F**), 100 µm; (**B**), 50 µm; (**A**,**D**,**H**), 10 µm; (**G**,**I**), 5 µm; (**J**–**L**), 1 µm.

SEM analysis of native muscle samples showed intact myofibers with densely packed myofibrillar elements within cells. Endomysial and perimysial structures, mainly composed of collagen bundles, were clearly visible. In decellularized samples from all the species, examined muscle cells were no longer visible. Endomysial collagen networks, composed of thin-fibrils in a mesh-like pattern, were preserved. Perimysial collagen was organized in thick bundles preferentially aligned along the axis of the previously present skeletal muscle fibers. Elastic components were also appreciable at higher magnifications, which also revealed that collagen bundles consisted of small fibrils with typical banding patterns (Figure 9).

**Figure 10:**
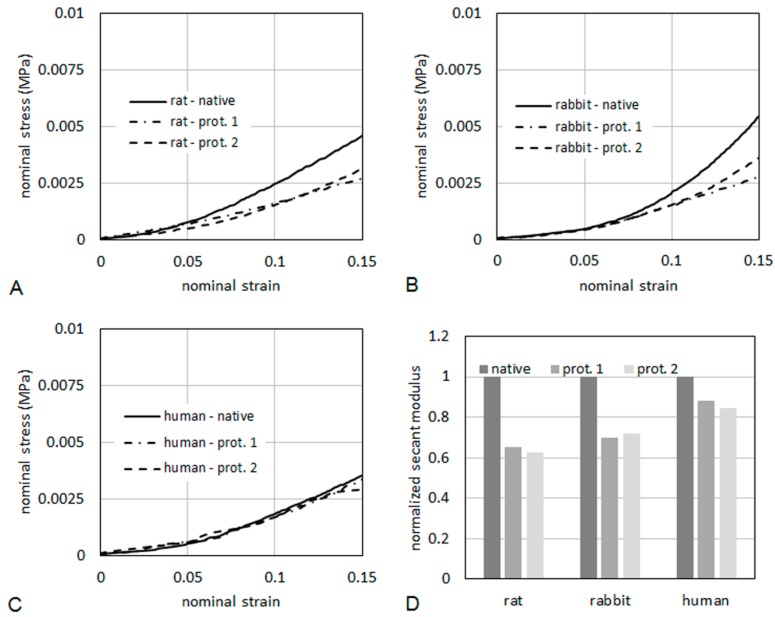
Nominal stress *vs.* nominal strain from tensile tests for rat (**A**), human (**B**) and rabbit (**C**). The secant elastic modulus at 10% of strain (**D**) is shown normalized to the secant modulus of the respective native tissue.

As it regards mechanical properties of the tissues, typical curves of nominal stress *vs.* nominal strain are reported in Figure 10, for specimens taken from rat (Figure 10A), rabbit (Figure 10B) and human (Figure 10C), respectively. The curves were limited to a maximum strain of 15%. The tissues were also compared in terms of secant elastic modulus (Figure 10D), defined as the stress/strain ratio at a specified level of strain. In the present case, the secant elastic modulus was evaluated at a strain of 10%. The secant modulus is shown normalized to the secant modulus of the respective native tissue. This makes a direct evaluation of the percentage reduction of the stiffness induced on the tissue by the decellularization process possible. After decellularization protocol 2, the normalized secant modulus was higher than 60% of native muscle in rats and rabbits, and higher than 80% in humans, demonstrating quite good preservation of the mechanical properties.

As it regards the results of surgical implantation, it must first be stressed that the human-derived scaffolds showed enough mechanical resistance to permit the suture with the native rabbit muscle structures. In the weeks after surgery, the graft prevented visceral herniation and did not show local signs of rejection, apart from mild inflammation ascribable to innate immune response. Rabbits did not show systemic signs of infection.

**Figure 11:**
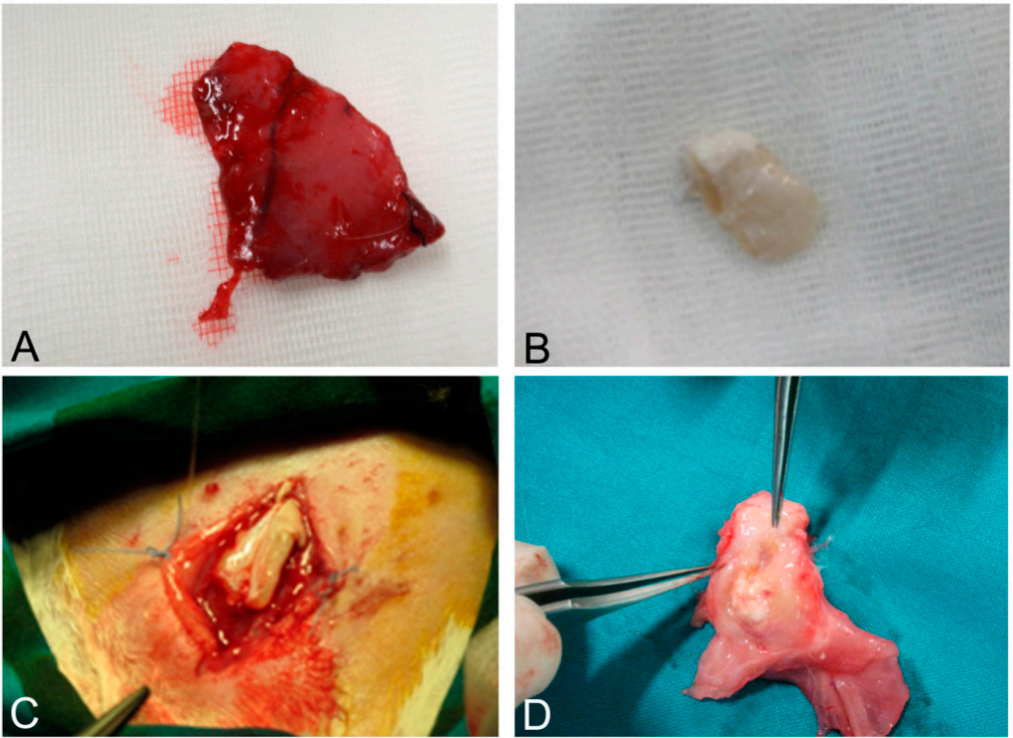

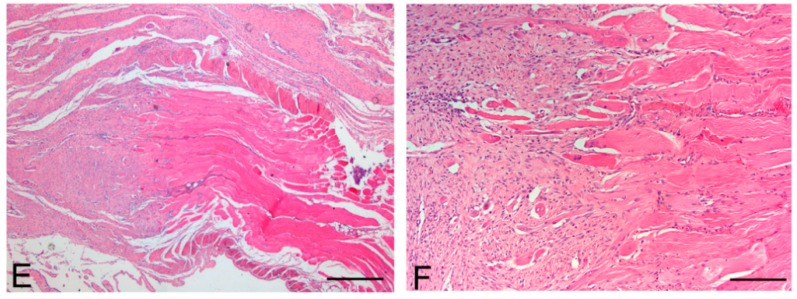
Native skeletal muscle of approximately 3 × 2 cm^2^ in size sampled from human abdominal rectus muscle (**A**); The same skeletal muscle sample after decellularization process (**B**); implant of skeletal muscle scaffold in the rabbit abdominal wall (**C**); Skeletal muscle scaffold explanted at sacrifice, three weeks after surgery (**D**); Histological analysis with hematoxylin and eosin staining, showing the biointegration between the scaffold and the native skeletal muscle tissue (**E**,**F**). Scale bars: (**E**), 300 µm, (**F**), 75 µm.

At sacrifice, scaffolds were well integrated with surrounding native muscle structures. Fistulas or mechanical breakdowns were absent. Macroscopically, the implanted system appeared pink, with signs of vascularization. Signs of granulomatous reaction were sometimes appreciable in the periphery of grafts, mainly at the level of absorbable sutures (Figure 11A–D).

At microscopic examination of the explanted grafts, most volume of the human-derived scaffolds was replaced by a fibrous connective tissue, showing fibroblast invasion and neo-vascularization. Morphometric evaluation showed a significant increase in the connective component (blue-staining in Azan-Mallory) with respect to both decellularized and native muscle samples, confirming the fibrotic response of the tissue (Figure 4). Some modest inflammatory infiltrates were also recognizable. Although morphometric evaluation in the context of the implanted scaffolds did not show an increase in muscle fiber content (red-staining in Azan-Mallory) with respect to decellularized materials (Figure 4), at the periphery of the implants, the native rabbit muscle fibers appeared in continuity with the human-derived scaffold and fibrous connective tissue, suggesting at least partial promotion of growth and migration of muscle fibers or progenitor cells (Figure 11E,F).

## 3. Discussion

In the present study, we confirmed the possibility of decellularizing human skeletal muscle in order to obtain a biologic scaffold that maintains the three-dimensional structure of the extracellular matrix. Ultrastructural analyses also demonstrated the preservation of collagen, elastic fibers, and proteoglycans, together with the selective removal of all cell structures. In particular, TEM analyses showed the preservation of the basal lamina of the muscle fibers, which could be particularly important for following *in vitro* and/or *in vivo* recellularization of the scaffold. Various biological scaffolds are commercially available derived from human tissues, such as skin and dermis (AlloDerm, Axis™ dermis, Bard^®^ Dermal Allograft), fascia lata (AlloPatch^®^, FasLata^®^, Suspend™) and pericardium (IOPatch™, IOP Inc., Costa Mesa, CA, USA) [27,28]. Decellularized omentum [25] and placental matrix [27,29,30,31] have also been proposed as tissue-engineered adipose substitutes. In the present study, we have shown that decellularized human skeletal muscles could also be used as a biologic scaffold. Human skeletal muscles were sampled from amputated limbs or from cadavers, both managed by the Body Donation Program of the Section of Human Anatomy, University of Padova [32]. We did not observe differences in the results of decellularization between amputated limbs and cadaveric samples, suggesting that both surgical and cadaveric materials could be used. The use of human extracellular matrix could favour the recellularization of the scaffold, with respect to animal-derived grafts, and prevent immune response.

In particular, in the present paper, we compared different decellularization protocols. In the literature, the first decellularization protocol was developed by Carlson and Carlson [33], who proposed a method involving several steps: skeletal muscle was first treated with chelants and detergents (EDTA and Triton X-100), to disrupt and remove the cell membrane, then it was exposed to enzymatic digestion (DNase) and it was finally treated with sodium deoxycholate to remove cellular debris. Following decellularization, protocols were implemented and refined through the introduction of freezing/thawing cycles and further chemical (e.g., SDS) and enzymatic (e.g., trypsin, latrunculin B) reactions [14,15,16,17,18,34,35]. Time of SDS incubation was suggested to be less than 72 h in order to avoid alterations of the extracellular matrix [13]. Additional treatments include the use of chloroform to remove the lipid components in the skeletal muscle [2,15,16].

In our study, two different decellularization protocols were used and compared: protocol 1 mainly involves sodium deoxycholate and DNase I; protocol 2 includes trypsin, EDTA, Triton and NH4OH. Protocol 2, which was stronger and involved different decellularization mechanisms, was more effective in removing the muscle fibers and cellular debris. Protocol 1 did not assure a complete decellularization either in human or animal samples. Morphometric analysis on sections stained with Azan-Mallory showed higher muscle fiber content in protocol 1 than in protocol 2, although a methodological limit of the analysis relies on the fact that the above staining is qualitative in nature, thus partially affecting technique reproducibility. Moreover, protocol 2 preserved the three-dimensional organization and ultrastructure of the extracellular matrix, providing a good balance between needs for removing cells and preserving the extracellular matrix.

The removal of DNA components is pivotal to avoid any adverse host response. Our results showed good removal of DNA content with both protocols but protocol 2, however, was more effective, assuring a concentration of dsDNA less than 10 ng per mg dry weight in all trials, consistently with literature references [22,23,24,25], and the absence of DNA fragments longer than 300 bp, in accordance with Gilbert *et al.* [26]. Moreover, any residual DNA is logically subject to enzymatic degradation after placement *in vivo* [26].

The scaffold itself showed the conservation of the three dimensional vascular network, suggesting the possibility of colonization of vessels by endothelial cells after *in vivo* implantation. This finding is of particular importance as previous papers have shown that scaffolds which had been seeded *in vitro* with endothelial cells demonstrated significantly greater vascularisation and tissue formation *in vivo* [36]. In the literature, implants of decellularized skeletal muscles have been reported in animals with contrasting results. In a previous study, a decellularized diaphragm muscle had been implanted in a rabbit with damage to the abdominal wall. After 40 days, microscopic examination did not show inflammatory reaction but fibroblast proliferation and capillary growth. Despite that, no migration of skeletal muscle cells was observed in the scaffold, which was mainly substituted by fibrous tissue [18]. These results were partially confirmed by Merritt *et al.* [2], who analyzed the implant of a muscle scaffold obtained from the decellularization of rat gastrocnemius. One month after the implant, an increased number of myofibers and endothelial cells was observed in the periphery of scaffold, suggesting a guiding role of the extracellular matrix components of the scaffold during the reparative process. Another study suggested that the transfer of an acellular skeletal muscle scaffold in a rat tibialis anterior resulted in an inflammatory reaction, composed of lymphocytes, granulocytes and macrophages, which was followed by tissue repair. In fact, immunofluorescence analysis revealed the presence of new myofibers inside the scaffold, resulting from migration of satellite cells [15]. A decellularized scaffold derived from the mouse latissimus dorsi has also been recently performed and implanted for biocompatibility in other mice. Inflammatory infiltration was mainly found after 10 or 20 days and then gradually decreased until postoperative day 30. In this case, no myofibril formation was seen [37]. To the best of our knowledge, this is the first study in which a human-derived scaffold obtained by decellularization of skeletal muscle was surgically implanted in an animal model. The biologic scaffold showed suitable mechanical properties for surgical implantation. The mechanical properties of the decellularized tissues were also compared with native muscles, showing good preservation of the normalized secant modulus, above all in human samples. From a surgical point of view, it was technically possible to suture the graft on the surrounding native muscle structures. In the three weeks after surgery, the implant was not rejected and histological analyses of the sample, after sacrifice, did not show such an inflammatory reaction to prevent scaffold integration, apart from mild inflammatory reaction due to innate immune response. Previous studies have shown the absence of inflammatory responses preventing rejection in implantation of animal decellularized skeletal muscle [38,39]. In the present paper, we demonstrated for the first time the biocompatibility of decellularized human skeletal muscle. Most of the volume of the scaffold was invaded by fibroblast, the three-dimensional architecture of the extracellular matrix being replaced by a fibrous connective structure, as was also confirmed by morphometric analyses. Conversely, at the periphery of the implant, continuity was appreciable between the human scaffold and the rabbit native muscle, with the scaffold components promoting growth and migration of the native muscle fibers, but recovery of skeletal muscle fiber population in the centre of implant was not achieved. Thus, human-derived scaffolds from skeletal muscle may be surgically implanted and may induce a reparative response (in absence of inflammatory reactions of such a degree to prevent integration), although recellularization by the near muscle cells and progenitors was not complete. However, it must also be considered that previous papers have not shown that the implant of a bladder acellular matrix device into a volumetric muscle loss defect in rat tibialis anterior is sufficient to promote a partial regeneration of the muscle loss even if the better functional recovery is achieved by the inclusion of muscle derived cells into the scaffold. This suggests that the delivery of acellular scaffolds could improve functional hypertrophy of the remaining muscle, promoting the force transmission across the scaffold [40]. Some reports have already considered the possibility to recellularize animal scaffolds with animal cells. It has been stressed that previous cell culture could not only promote the reparative process of injuries but also enhance the regenerative response of the muscle with the formation of new myofibers and vascular channels [2,40,41]. Effectively, when murine C2C12 myoblasts have been cultured on tissue culture surfaces coated with skeletal muscle scaffold, the levels of proliferation and differentiation have increased, stressing the role of collagenic components [34,41]. Some researchers implanted *in vivo* a scaffold that was recellularized before with skeletal muscle cells *in vitro*. The analyses of the patch after the implant in the donor site revealed the presence of mature skeletal muscle fibers confirming the effectiveness of the recellularized scaffold not only in a reparative process but also in a regenerative response [16,19,41]. In this way, scaffolds could promote the healing of the muscle damage with substitutes of structure and composition quite similar to native muscle.

Progenitors of human muscle cells have also been previously used for cellularization of artificial scaffolds. For instance, in 2005, Riboldi and colleagues positively verified the capability of primary human satellite cells to adhere, proliferate and fuse together in the biodegradable block copolymer (DegraPol^®^, ab medica, Lainate, Milan, Italy) [42].

Future evolution of our study will involve the recellularization of the human scaffold with human derived cells. Preliminary analyses could involve commercially available Human Skeletal Myoblasts, in order to evaluate the suitability of the scaffold for recellularization. Further steps could include autologous recellularization with human cells with myogenic potential. Many different cell types have been identified, as potential candidates for cell therapeutic approaches for muscle degeneration [43], which could be also suitable for scaffold recellularization. Satellite cells are quiescent unipotent stem cells located beneath the basal lamina of adult skeletal muscle fibers [43,44,45,46]. In response to muscle damage, these cells can proliferate and give rise to myogenic precursors able to fuse and form new muscle fibers [43,44,46]. Satellite cells could be isolated from fresh skeletal muscle biopsies and expanded *in vitro* with the addition of autologous serum to improve skeletal muscle myofiber differentiation and growth [45,47]. The *in vivo* injection of satellite cells, however, has also revealed some limitations due to reduced migratory ability and myogenic capacity when expanded *in vitro* [43]. Other potentially useful cell types are the skeletal myogenic precursors, a sub-type of satellite cells, the mesoangioblasts, a new class of stem cells associated with vasculatures which has also been isolated from human adult skeletal muscle, and the fibro-adipogenic precursors [43]. A growing interest has also arisen about the therapeutic perspective offered by the induced pluripotent stem (iPS) cells. Preclinical evidences have shown that myoblasts and mesenchymal cells derived from human iPS could efficiently fuse with mature muscle fibers [48,49] and improve the performances of engrafted muscles [50]. Autologous adipose-derived stem cells may also potentially be used for re-cellularization of skeletal muscle scaffolds. These cells have the capability to differentiate along different lineage, comprising the myogenic one [51], and can easily be derived from liposuctioned fat from the same patient. After a first *in vitro* recellularization phase, further cell invasion will derive *in vivo* after implantation, although these aspects must be addressed in future works.

Further analyses will also have to address the capability of bio-engineered muscles involving human scaffolds to be reinnervated. The development of a functional nerve-muscle interface is a pivotal issue to increase the usefulness of engineered muscles in a clinical setting. However, promising works have been published regarding *in vitro* [52,53] and *in vivo* [36,54,55,56,57] neurotisation of engineered muscles, with *de novo* formation of neuromuscular junctions.

As it regards future clinical applications of these scaffolds, it must be considered that in the present study they were not terminally sterilized whereas specific sterilization will be necessary prior to use in a clinical setting, requiring further confirmations of structural, compositional and mechanical properties of the material.

In conclusion, histological (H&E, Azan-Mallory, Van Gieson, Alcian blue, DAPI) and ultrastructural (TEM, SEM) methods, together with DNA extraction and quantification techniques, have demonstrated the possibility to efficiently decellularize human skeletal muscle, with total removal of cellular materials and preservation of the three-dimensional network of extracellular matrix. Comparative analysis of different protocols in different species (rat, rabbit, human) showed better results with the involvement of trypsin-EDTA and Triton X-NH4OH. Surgical trials of scaffold implantation provided good results in terms of mechanical properties and biointegration, although mainly through reparative mechanisms more than regenerative ones. Preliminary *in vitro* recolonization by autologous cells will have to be tested in the future to enhance muscle regeneration. Our study showed that biologic scaffolds from human skeletal muscle, derived from surgical specimens or cadavers, may be promising in reconstructing/regenerative approaches to skeletal muscle losses and may be added to other human-derived scaffolds (dermis, fascia lata, pericardium, placental matrix, omentum).

## 4. Materials and Methods

### 4.1. Muscle Samples

The present study was approved by the Italian Public Health Office regulations and by the Ethics Committee of the University of Padua. Procedures on animals were performed at the Experimental Surgery Centre and in the Department of Molecular Medicine of the University of Padua. Animal muscle samples of approximately 3 × 2 cm^2^ in size were taken from adult Sprague-Dawley rats and “New Zealand White” rabbits. Human skeletal muscles of approximately 3 × 2 cm^2^ in size were sampled from amputated limbs or cadavers, both managed by the Body Donation Program of the Section of Human Anatomy, University of Padova [58,59], according to European, Italian and regional guidelines [32,60]. In both animals and humans, tibialis anterior and abdominal rectus muscles were sampled. Four samples were taken from each species and each muscle type.

### 4.2. Skeletal Muscle Decellularization

Muscle samples of rats, rabbits and humans were decellularized by the application of two different protocols, in order to test differences in their effectiveness. For each species, the specimens subjected to morphological/morphometric analyses included eight native samples, eight samples decellularized with protocol n.1, adapted from decellularization protocol reported in literature [61], and eight samples decellularized with protocol n.2, adapted from Sheridan’s protocol [62]. Human samples were taken from 4 male and 4 female subjects, with a mean age (±Standard Deviation) of 70.5 (±8) years.

Protocol n.1: samples were washed in phosphate-buffered saline (PBS) with 1% antifungal and antibiotic solution (ABAM, A5955, Sigma-Aldrich, Milan, Italy). They were incubated with ultrapure water for 72 h at 4 °C, then with 4% sodium deoxycholate (Sigma-Aldrich) for 4 h at 4 °C and then with 2000 K DNase I (Sigma-Aldrich) for 2 h at 37 °C. Samples were kept in agitation for all the procedures. This process was repeated four times. At the end of the processing, the decellularized matrices were rinsed three times in PBS.

Protocol n.2: samples were washed with ultrapure water for 24 h at 4 °C, then incubated for 1 h with 0.05% trypsin-0.02% EDTA at 37 °C and, after a washing in PBS, incubated with 2% Triton X-100-0.8% NH_4_OH for 72 h at 4 °C in continuous agitation. Samples were washed with ultrapure water for 48 h. At the end of the processing, the decellularized matrices were rinsed three times in PBS.

### 4.3. Morphological and Morphometric Analyses

Morphological and ultrastructural analyses, together with DNA quantification, were performed both in native muscles and decellularized samples. Samples were fixed in 10% neutral buffered formalin for 24 h, embedded in paraffin and sectioned at 5–6 μm thickness. Representative samples were taken from various regions of the decellularized scaffolds, including the most central regions, as well as the exterior surfaces. The following histochemical stainings were performed: Haematoxilin and Eosin (H&E), Azan-Mallory and Van Gieson.

Quantitative morphometric evaluations of the different tissue components were performed with the help of ImageJ software [63], freely available at http://rsb.info.nih.gov/ij/, by applying image analysis procedures previously detailed in works of our group [64,65,66]. In particular, the contents of skeletal muscle fibers and connective tissue were evaluated in terms of percentage areas stained in red/violet and blue, respectively, with Azan-Mallory. To morphometrically evaluate the above components, the ImageJ software was implemented by a Macro. Pictures of sections stained with Azan-Mallory were acquired. The colours of the picture were analysed, displaying histograms of the distribution of hue, saturation and brightness. The intervals of colours corresponding to the above tissue components in the different stainings were manually selected and maintained for all correspondent analyses. The selected intervals of colours were converted into white and all the other colours into black. To facilitate the process of evaluation, the white and black colours were inverted. On the processed images, the areas corresponding to skeletal muscle fibers or connective tissue were selected, and the percentages of tissue components, represented by the colour white, were automatically measured (Figure S3).

### 4.4. DAPI Staining, DNA Extraction and Quantification

Four samples for each species and protocol were also frozen in OCT compound for 4′,6-diamidino-2-phenylindole (DAPI) staining. Sections were fixed in acetone for 15 min at room temperature, washed in PBS for 10 min, incubated for 1 min in DAPI diluted 1:500 in methanol, washed twice in PBS, covered, and then examined under a fluorescent microscope.

Four samples for each species and protocol were also frozen on dry ice and stored at −80 °C for DNA extraction and quantification. Genomic DNA was extracted with the DNeasy^®^ Blood & Tissue Kit (QIAGEN, Milan, Italy). Briefly, tissue was cut into small pieces, placed in a 1.5-mL microcentrifuge tube, and incubated with 20 μL of proteinase K for 56 °C until completely lysed. To collect genomic DNA, 200 μL of first Buffer AL are added and then 200 μL ethanol (96%–100%). The mixture was pipetted into a DNeasy Mini spin column placed in a 2-mL collection tube and centrifuged at 8000 rpm for 1 min; the flow-through was discarded. To wash DNA, spin column was filled in with 500 μL of Buffer AW1, centrifuged for 1 min at 8000 rpm, added with 500 μL of Buffer AW2, and centrifuged for 3 min at 14,000 rpm. The DNA was eluted by adding 200 μL of Elution Buffer and centrifuged for 1 min at 8000 rpm.

Total DNA concentration was measured with a spectrophotometer (NANODROP 1000, Techno Scientific, CELBIO, Milan, Italy) by reading the optical density (O.D.) at γ = 260 nm, corresponding to the maximum absorption of nitrogenous bases.

To determine DNA fragment size, samples were separated by electrophoresis on a 3% NuSieve GTG Agarose gel (Lonza, Rockland, ME, USA) with GelRed (Biotium, Hayward, CA, USA) at 60 V for 1 h stained and visualized with ultraviolet transillumination [26].

### 4.5. Transmission Electron Microscopy (TEM)

Two specimens for each species and protocol were studies with TEM. Samples were fixed in 3% glutaraldehyde (Serva Electrophoresis, Heidelberg, Germany) in 0.1 M PBS, post-fixed in 1% osmium tetroxide (Agar Scientific Elektron Technology, Stansted, UK) in 0.1 M PBS, dehydrated in a graded alcohol series and embedded in Epoxy resin. Ultrathin 60 nm-thick sections were cut with an ultramicrotome (LKB-8800 Ultratome III, LKB Produkter, Stockholm, Sweden) collected on 300-mesh copper grids, counterstained with 2% uranyl acetate and then with Sato’s lead. Specimens were observed by a Hitachi H-300 Transmission Electron Microscope (Hitachi, Krefeld, Germany).

### 4.6. Scanning Electron Microscopy (SEM)

Two further specimens for each species and protocol were studied with SEM. Samples were fixed in 3% glutaraldehyde in 0.1 M PBS pH 7.0 overnight at 4 °C. They were dehydrated with ethanol washing at the concentration of 20%, 40%, 60%, 80% and 100% for 1 h at room temperature for each concentration and then they were submitted to Critical Point Dryer (Bal-tec CDP 010, Bal-Tec GmbH, Pfäffikon, Switzerland) for 1 h. Specimens were stuck on carbon double-side tape with silver paste, coat with gold alloy (Edwards S150A, sputter coating unit, Edwards, Crawley, UK) and analyzed with JSM-6490 SEM (JEOL, Tokio, Japan).

### 4.7. Mechanical Testing of the Tissues

Uniaxial tensile tests under control of displacement were developed to evaluate stiffness and strength properties of native and decellularized tissue. One sample was tested for each species and each protocol. The testing was performed in the Planar Biaxial TestBench Test Instrument Bose^®^ Electro-Force (Electro-Force Systems Group, Eden Prairie, MN, USA) at room temperature (20 ± 1 °C) and keeping the specimen hydrated by means of a continuous pipetting with saline solution. The specimens had rectangular shape with average width of 4.8 mm and gauge length of 10 mm. The test protocol consisted of five tensile loading/unloading cycles up to 5% of strain, followed by a tensile loading up to the failure. An elongation rate of 0.01/s was applied in all the cycles. The apparatus recorded the tensile force *vs.* elongation of the specimen. The mechanical properties of the tissue were evaluated in terms of nominal strain (elongation normalized to initial gauge length) and nominal stress (tensile force normalized to initial transversal area) from the last loading cycle. Width and thickness of the specimens were evaluated by means of digital images analysis.

### 4.8. Implantation of Human Graft in Rabbit Recipient

Four human decellularized scaffolds of about 1.5 × 2 cm^2^ in size, derived from abdominal rectus muscle and treated with protocol 2, were also implanted in four receiving rabbits, in which an artificial surgical resection of the abdominal rectus muscle of approximately 1.5 × 2 cm^2^ in size had been produced, causing herniation of the abdominal viscera.

Rabbits were subjected to anaesthesia by intramuscular 0.6 mL/kg of tiletamine hydrochloride and zolazepam. General anaesthesia was maintained during the surgical intervention by inhalation mask in isoflurane. The parameters and the state of anaesthesia were monitored by the staff through control of respiratory excursions, response to painful stimuli and corneal reflexes. Regional subcutaneous anaesthesia with lidocaine 1% was performed at the incision site.

Once anaesthesia was considered satisfactory, the surgical procedure was performed in sterile conditions where both the groin and the abdominal region had been washed with chlorhexidine solution and disinfected with iodopovidone alcohol. An oblique incision of 5 cm was practiced at the abdominal region and subsequently extended proximally, marking the limits of the abdominal flap. Adipose tissue and fascia were removed to permit the identification of the rectus muscle of the abdomen and the dissection of the flap was performed by the use of an operating microscope (Carl Zeiss Inc., OPM1, Carl Zeiss Inc., Oberkochen, Germany) and microsurgical instrumentation. Rectus muscle of 1.5 × 2 cm^2^ in size was resected. In the same surgical procedure, a scaffold of 1.5 × 2 cm^2^ in size derived from a human sample was placed at the site of the wound, through the use of the surgical microscope. To promote adhesion to the native muscle, the transplanted flap was sutured through the use of 5–0 absorbable glyconate synthetic monofilament thread around the perimeter.

At the end of the microsurgical operation, the recipient rabbit was subjected to antibiotic prophylaxis with 5 mg/kg of enrofloxacin maintained thereafter for a further 7 days. In these animals, 5 mg/kg of tramadol was subsequently administered to relieve pain, repeated twice in the next 48 h. In the postoperative phase, the animal was observed daily for 3 weeks and then sacrificed.

### 4.9. Explanation and Analysis of the Implanted Human Scaffold

After sacrifice, an oblique incision of 5 cm was practiced at the abdominal site and subsequently proximally extended in the limits of the abdominal flap. After the identification of the implant, all of the tissue complex was resected, including portions of surrounding native tissue.

The sample was fixed in 10% neutral buffered formalin for 24 h, embedded in paraffin, sectioned at 5–6 μm thickness and analysed with the above histological stainings. Morphometric evaluation of the muscle fiber content and connective tissue was also performed in implant samples, according to methods exposed in Section 4.3.

### 4.10. Statistical Analysis

Statistical analysis was performed by one-way analysis of variance (ANOVA) and Tukey’s multiple comparison test. *p* < 0.05 was considered to be statistically significant. Statistical calculations were carried out by Prism 3.0.3 (GraphPad Software, San Diego, CA, USA).

## Supplementary Materials

Supplementary materials can be found at http://www.mdpi.com/1422-0067/16/07/14808/s1.
