# Supplementary material for: Decellularized Human Skeletal Muscle as Biologic Scaffold for Reconstructive Surgery
Source: Int J Mol Sci. 2015 Jul 1;16(7):14808–31. doi: 10.3390/ijms160714808 (PMC4519873; doi:10.3390/ijms160714808)
Supplement: Supplementary file 1 [file ijms-16-14808-s001.pdf]

# Supplementary Information

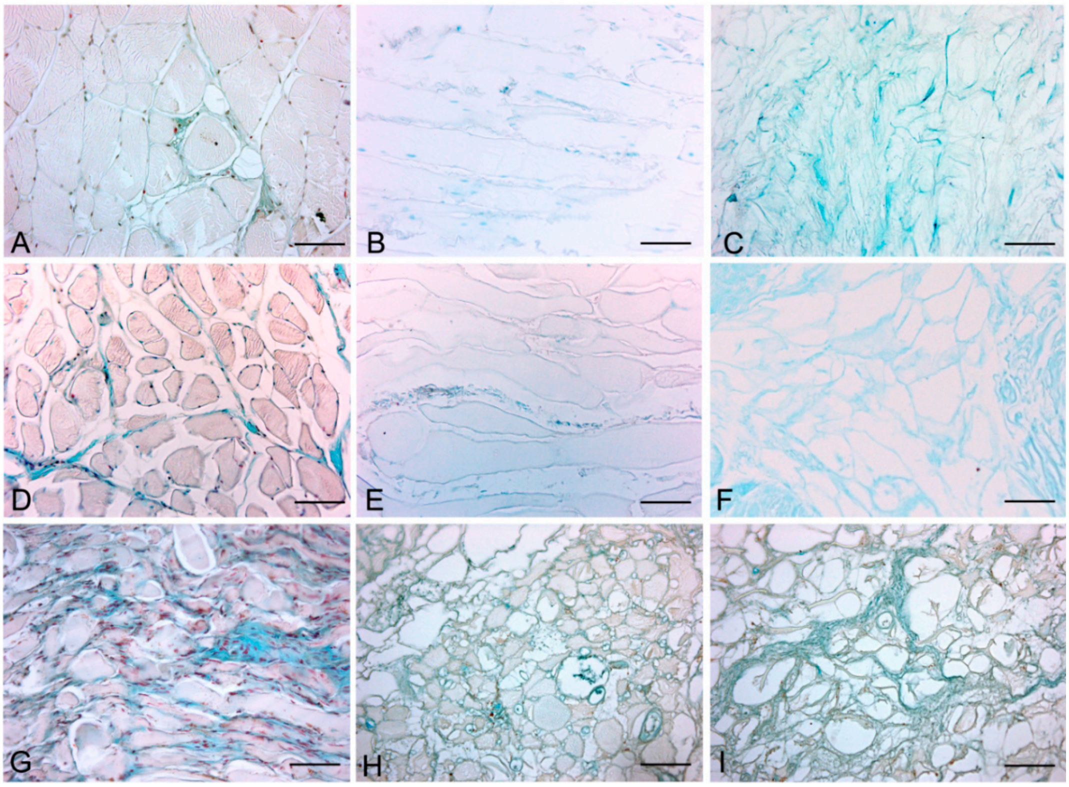


**Figure S1.** Representative sections of rat (**A**–**C**), rabbit (**D**–**F**) and human (**G**–**I**) skeletal muscle, before decellularization (**A**,**D**,**G**), after decellularization protocol 1 (**B**,**E**,**H**) and after protocol 2 (**C**,**F**,**I**), showing the persistence of blue-staining sulfated glycosaminoglycans in both decellularization protocols (Alcian blue, pH 1). Scale bars: (**A**–**I**): 75 µm.

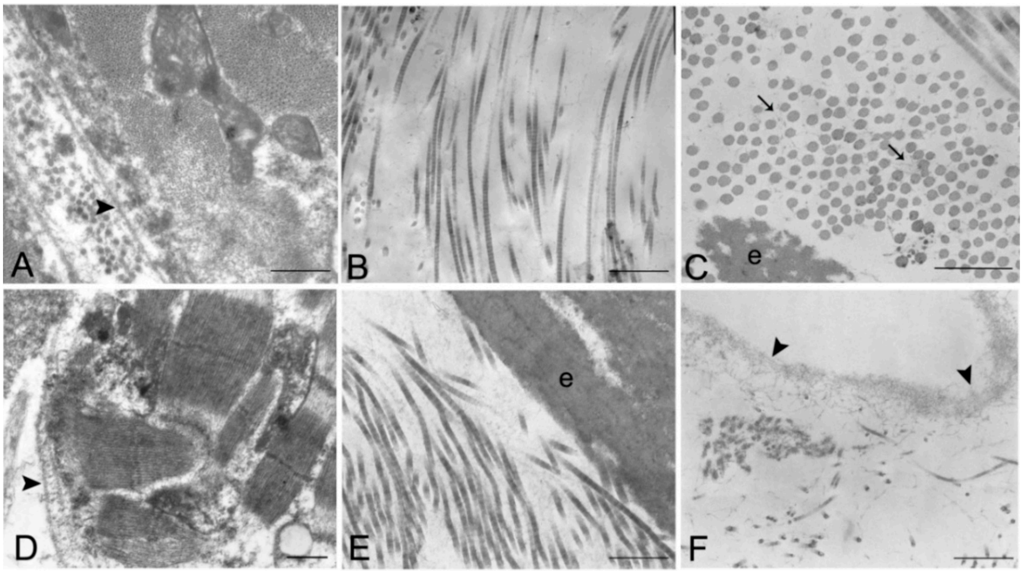


**Figure S2.** Transmission electron microscopy of skeletal muscle of rat (**A**–**C**) and rabbit (**D**–**F**) before decellularization (**A**,**D**) and after decellularization protocols 1 (**B**,**E**) and 2 (**C**,**F**); Note the preservation of basal lamina (arrowheads in (**A**,**D**,**F**)) of muscle fibers, and the maintenance of the ultrastructure of collagen, elastic fibers (e) and proteoglycans (arrows in (**C**)) in the extracellular matrix. Scale bars: (**A**–**F**), 0.5 µm.

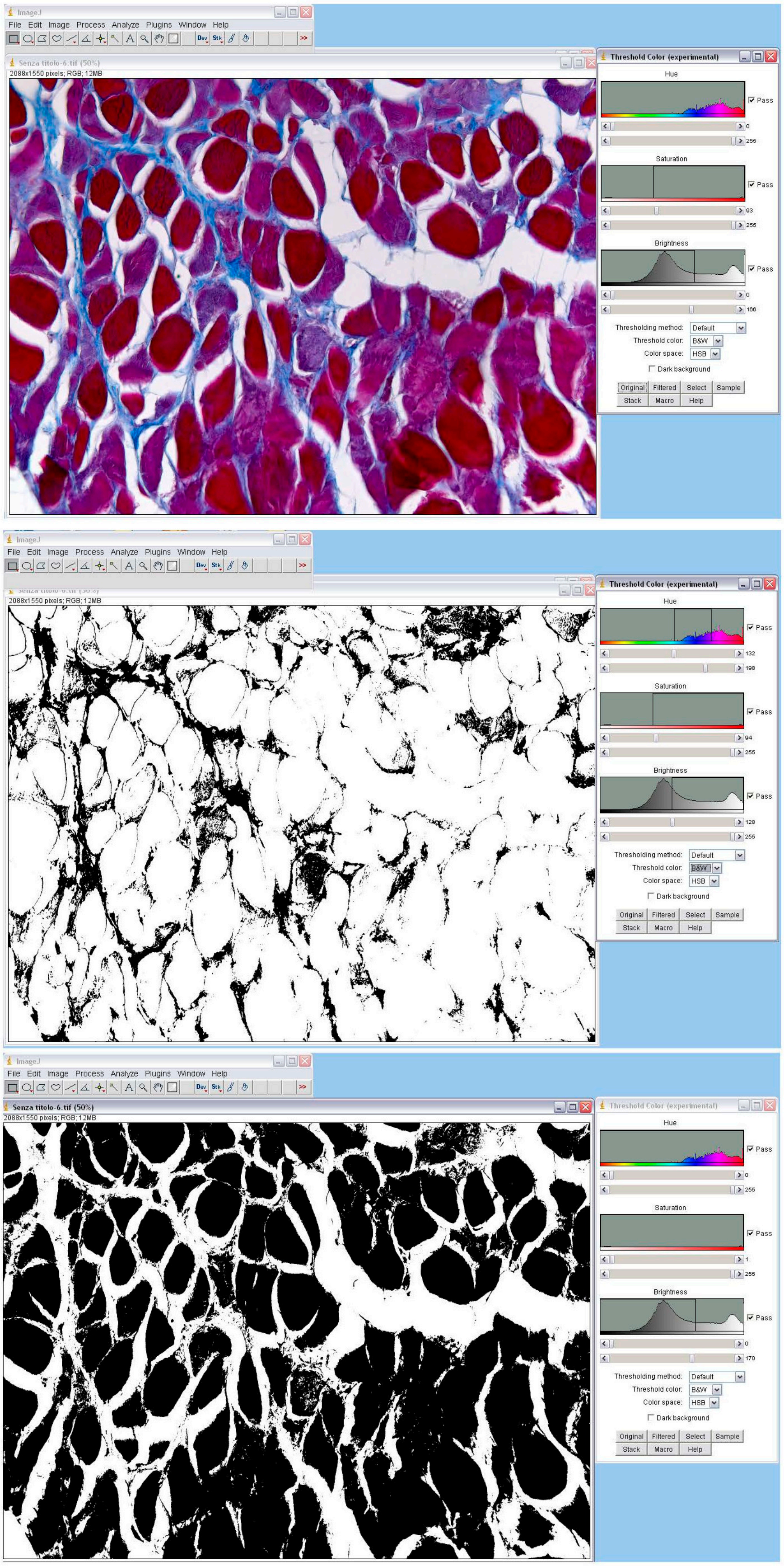


**Figure S3.** Morphometric method of quantification of muscle fiber content and connective tissue in azan-Mallory stained sections. Pictures of sections stained with azan-Mallory were acquired. The colours of the picture were analysed, displaying histograms of the distribution

of hue, saturation and brightness. The intervals of colours corresponding to the above tissue components in the different stainings were manually selected and maintained for all correspondent analyses (**higher** panel). The selected intervals of colours were converted into white and all the other colours into black (**intermediate** panel). To facilitate the process of evaluation, the white and black colours were inverted. On the processed images, the areas corresponding to skeletal muscle fibers or connective tissue were selected, and the percentages of tissue components, represented by the colour white, were automatically measured (**lower** panel).
